# Inter-limb weight transfer strategy during walking after unilateral transfemoral amputation

**DOI:** 10.1038/s41598-021-84357-9

**Published:** 2021-02-26

**Authors:** Ryo Amma, Genki Hisano, Hiroto Murata, Matthew J. Major, Hiroshi Takemura, Hiroaki Hobara

**Affiliations:** 1 Artificial Intelligence Research Center, National Institute of Advanced Industrial Science and Technology (AIST), AIST Waterfront 3F, 2-3-26, Aomi, Koto-ku, Tokyo, 135-0064 Japan; 2 Department of Mechanical Engineering, Tokyo University of Science, Chiba, Japan; 3 Department of Systems and Control Engineering, Tokyo Institute of Technology, Tokyo, Japan; 4 Department of Physical Medicine and Rehabilitation, Northwestern University, Chicago, IL USA; 5 Jesse Brown VA Medical Center, Chicago, IL USA

**Keywords:** Biomedical engineering, Rehabilitation

## Abstract

Although weight transfer is an important component of gait rehabilitation, the biomechanical strategy underlying the vertical ground reaction force loading/unloading in individuals with unilateral transfemoral amputation between intact and prosthetic limbs remains unclear. We investigated weight transfer between limbs at different walking speeds in 15 individuals with unilateral transfemoral amputation and 15 individuals without amputation as controls, who walked on an instrumented treadmill. The normalized unloading and loading rates were calculated as the slope of decay and rise phase of the vertical ground reaction force, respectively. We performed linear regression analyses for trailing limb’s unloading rate and leading limb’s loading rate between the prosthetic, intact, and control limbs. While loading rate increased with walking speed in all three limbs, the greatest increase was observed in the intact limb. In contrast to the other limbs, the prosthetic limb unloading rate was relatively insensitive to speed changes. Consequently, the regression line between trailing prosthetic and leading intact limbs deviated from other relationships. These results suggest that weight transfer is varied whether the leading or trailing limb is the prosthetic or intact side, and the loading rate of the leading limb is partially affected by the unloading rate of the contralateral trailing limb.

## Introduction

Lower limb amputation often leads to reduced physical activity levels^1^, which can result in weight gain^2^, depression onset^3^, and increased risk of cardiovascular and other chronic diseases^4,5^. In turn, higher mobility has been linked to improved satisfaction and quality of life in patients after major lower limb amputation^3,6,7^, making mobility restoration a priority in prosthetic gait training for individuals with lower limb amputation. A recent study highlighted the importance of adequate prosthetic gait rehabilitation for patients with major lower limb amputation to regaining mobility^8^. Therefore, the quantification and evaluation of gait biomechanics in individuals with lower limb amputation is essential for delivering objective, targeted improvements in rehabilitation strategies and prosthetic design to maximize long-term mobility outcomes.

Individuals with unilateral transfemoral amputation (UTFA) have shown worse functional outcomes compared to their transtibial-level counterparts^8–10^. Further, studies have demonstrated that individuals with UTFA commonly suffer from disabilities including knee osteoarthritis^11^ or back pain^12^ that are secondary to the repetitive loading during step-to-step inter-limb body weight transfer. More generally, a previous study also suggested that surface fissures in articular cartilage may increase when the cartilage is subjected to cyclic loading^13^, and so such repetitive loading may lead to degenerative arthritis^14^. Therefore, the joint pain or degeneration often observed in individual with UTFA may be caused by increased loading on their intact limb^15^. Although evaluating the relationships between limb loading dynamics during walking and secondary musculoskeletal conditions may help inform rehabilitation strategies^16^ and prosthesis design^17^, the biomechanics underlying alternate weight loading/unloading between the intact and prosthetic limbs during walking in persons with UTFA have not yet been fully characterized.

As the transfer of weight between limbs (trailing limb unloading, leading limb loading) during the double support phase of walking occurs over a duration of time, characterization of weight transfer biomechanics requires consideration of both the temporal and magnitude aspects of loading. The unloading rate (ULR), defined by the change in vertical ground reaction force (vGRF) during push-off, may be used to characterize the unloading profile of the trailing limb^18^. The ULR is often calculated as the average slope from the second peak of vGRF to toe-off with units of N/s or the normalized units of body weight/s (BW/s)^19–22^. Likewise, the loading rate (LR), defined by the change in vGRF during load acceptance following foot initial contact, may be used to characterize the loading profile of the leading limb^23^. Importantly, evidence suggests that greater LR may be associated with elevated risk of musculoskeletal injuries^24,25^. The LR is generally calculated as the average slope from initial contact to the first peak of vGRF with units of N/s or the normalized units of BW/s^19–23^. Additionally, the ULR and LR of the trailing and leading limb, respectively, occur almost simultaneously during the double-support phase but are not necessarily equal in magnitude. The first and second peaks of vGRF in individuals without amputation were reported to increase by a similar amount across a range of walking speeds^26^. Consequently, the ULR and LR in individuals without amputation tend to increase with walking speeds^18,23^, indicating a speed-dependent unloading/loading strategy. However, the unloading/loading strategy during gait in individuals with UTFA may be different than those without amputation as reflected in observed differences in GRFs and limb mechanical work, a product of GRFs and body center of mass velocity^27^. For instance, the first peak of vGRF in individuals with UTFA have been reported to increase by a greater amount with increased walking speed in the intact limb than the prosthetic limb^15^. Additionally, Bonnet et al. demonstrated that mechanical work generated by each individual limb on the body center of mass during push-off of step-to-step transitions was considerably smaller for the prosthetic limb and greater for the intact limb in comparison to the limbs of individuals without amputation^27^. Importantly, the authors also demonstrated that the prosthetic limb in individuals with UTFA was unable to increase push-off mechanical work with increased walking speed as was demonstrated by the intact limb or the limbs in individuals without amputation^27^. Further, weak prosthetic limb push-off work may lead to increased collision work and first peak of vGRF in the intact limb^28^. While these differences may suggest adjustments to ULR and LR in persons with UTFA, little is known about the ULR and LR relationship in this cohort across a range of walking speeds. The ULR and LR relationship may represent the inter-limb weight transfer strategy and provide insight into the underlying coordination during walking between intact and prosthetic limbs in individuals with UTFA. Given the importance of inter-limb coordination in mechanical energy exchange to maintain efficient forward ambulation^27^, such characterization might help understand limitations in motor strategies in persons with UTFA and inform targeted rehabilitation interventions.

The aim of this study was to investigate the weight transfer strategy between intact and prosthetic limbs across a range of walking speeds in individuals with UTFA. To this end, we compared the following relationships between the ULR and LR, namely, the relationship between the ULR of the prosthetic limb (ULR_prosthetic_) and LR of the intact limb (LR_intact_), the relationship between the ULR of the intact limb (ULR_intact_) and the LR of the prosthetic limb (LR_prosthetic_), the ULR and LR of the control limb (ULR_control_ and LR_control_, respectively) from individuals without UTFA. As individuals with UTFA have morphologically and functionally asymmetric limbs, we hypothesized that weight transfer strategies as reflected by the ULR and LR would be dependent on which limb was in the trailing and leading position. We also expect that with increasing speed, the ULR and LR relationships in individuals with UTFA would deviate from that in individuals without amputation.

## Methods

### Participants

A convenience sample of fifteen individuals with UTFA (11 males, 4 females; age [mean ± standard deviation], 30 ± 8 years; height, 1.65 ± 0.09 m; mass, 65.7 ± 15.7 kg; time since amputation, 13 ± 9 years) participated in this study (Table 1). The amputation etiologies included cancer, trauma, sarcoma and congenital. In order to investigate the weight transfer strategy across a range of walking speeds, the recruitment criteria for prosthesis users were the following:

1. User of a unilateral transfemoral or knee-disarticulation prosthesis;
2. Functional Classification Level of K-3 or above and able to ambulate without using external aid or assistance;
3. Without confounding neurological or orthopedic issues throughout the body.

**Table 1:** Participants characteristics, where the weight includes any prosthetic component.

Participant	Sex	Age (years)	Height (m)	Mass (kg)	Time since amputation (years)	Prosthetic knee unit	Prosthetic feet	Amputated limb	Cause of amputation
**Individual with UTFA**									
1	M	23	1.68	58.3	20	3R80	Valiflex	Left	Cancer
2	M	27	1.75	71.0	6	3R80	1C64 Triton	Right	Trauma
3	M	34	1.61	61.4	21	3R95	Valiflex	Left	Sarcoma
4	M	17	1.77	84.0	3	NK-6	Triton	Right	Congenital
5	M	30	1.70	70.3	21	3R106	Valiflex	Right	Sarcoma
6	M	29	1.65	70.1	8	3R80	1C61 Triton	Right	Trauma
7	M	31	1.72	64.0	8	Mauch knee	Elation	Right	Sarcoma
8	M	36	1.61	60.1	18	3R106	Triton	Right	Trauma
9	M	42	1.75	111.1	25	3R80	Dyna Trek	Right	Trauma
10	M	42	1.70	75.4	32	3R80	Hilander	Right	Trauma
11	M	43	1.67	57.6	8	3R106	Triton	Left	Cancer
12	F	21	1.49	47.5	10	3R106	Total Concept	Right	Sarcoma
13	F	20	1.56	56.4	6	Total knee	Valiflex xc	Right	Trauma
14	F	21	1.52	52.1	13	3R106	Elation	Left	Sarcoma
15	F	27	1.54	46.4	1	3R60	RS2000 Runway	Right	Trauma
Mean		30	1.65	65.7	13				
SD		8	0.09	15.7	9				

Control									
1	M	22	1.70	63.1					
2	M	26	1.72	71.6					
3	M	25	1.60	61.9					
4	M	31	1.77	88.6					
5	M	28	1.78	72.1					
6	M	21	1.66	65.6					
7	M	32	1.71	80.5					
8	M	34	1.62	79.4					
9	M	38	1.70	79.6					
10	M	49	1.76	74.6					
11	M	51	1.67	65.3					
12	F	21	1.57	58.3					
13	F	23	1.59	54.2					
14	F	23	1.61	62.5					
15	F	28	1.63	52.9					
Mean		30	1.67	68.7					
SD		9	0.07	10.1					

*UTFA* unilateral transfemoral amputation, *M* male, *F* female, *SD* standard deviation.

All individuals with UTFA used their own mechanical prosthetic knees and feet (Table 1). We also recruited 15 individuals matched by sex, age, height, and weight without amputation to establish a control group (Table 1; 11 males, 4 females; age, 30 ± 9 years; height, 1.67 ± 0.07 m; mass, 68.7 ± 10.1 kg). Before the experiment, all the participants provided written informed consent as approved by the local ethics committee. The study was approved by the Institutional Review Board of our institution (Environment and Safety Headquarters, Safety Management Division, National Institute of Advanced Industrial Science and Technology) and conducted in accordance with the guidelines of the 1983 Declaration of Helsinki.

### Experimental procedures

**Figure 1:**
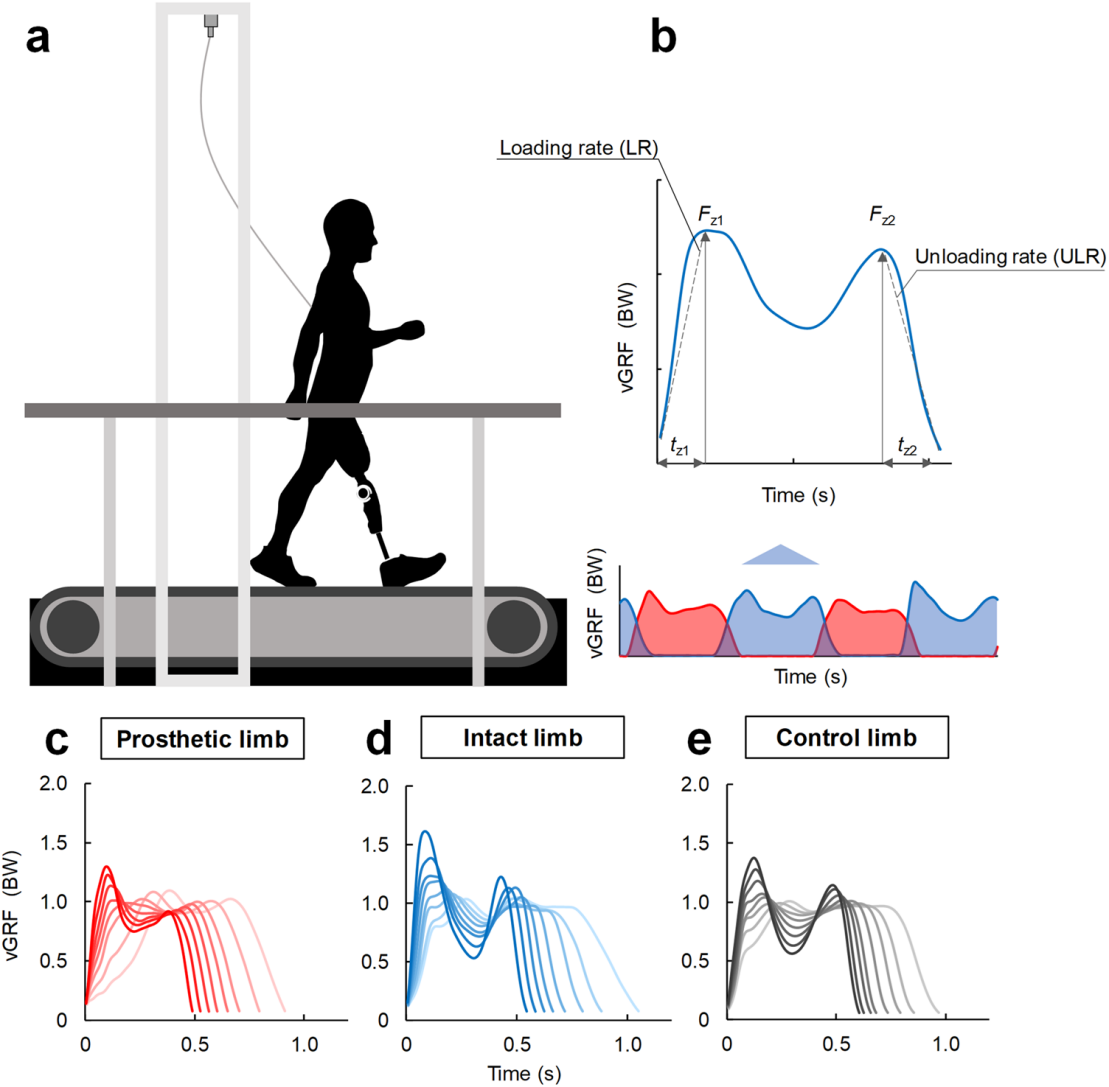
(**a**) Diagram of split-belt instrumented treadmill with handrails used in this study. A safety harness was attached to each participant to prevent falls during the experiments. (**b**) LR and ULR were calculated as the positive and negative vGRF slopes during walking, respectively. (**c**–**e**) vGRF recorded from a representative participant in the intact and prosthetic limbs and from a control individual. More intense color indicates higher walking speed from 2.0 to 5.5 km/h with increments of 0.5 km/h. BW, body weight. Figure was created in Microsoft Power Point for Office 365 MSO (16.0.12527.21378).

Prior to data collection, each participant walked on a split-belt force-instrumented treadmill (FTMH-1244WA, Tec Gihan, Kyoto, Japan; Fig. 1a) for at least 7 min to habituate as suggested by accommodation studies^29,30^. During the habituation period, participants experienced all experimental speeds (40–50 s for each), and we confirmed that they were able to walk at each speed. Following a subsequent rest period, participants walked on the instrumented treadmill at 8 speeds (2.0–5.5 km/h with increments of 0.5 km/h) for 30 s per speed. Through inspections of real-time data collection and video recordings, we confirmed that no participant held the handrails during data collection. In addition, the safety harness and rope were sufficiently slacked, and consequently, it did not reduce weight bearing during walking. The participants were allowed rest periods between trials as requested to minimize fatigue effects.

### Data collection and analysis

Consecutive steps within the boundaries of each belt at each speed were analyzed. Valid steps (i.e., those when the feet remained within the boundaries of individual belts) were confirmed through visual inspection of the vGRF time series plots. We averaged these steps to determine the representative values at each speed in each limb. On average, 27 ± 7 consecutive steps were analyzed per limb (i.e., prosthetic, intact, and right limbs in control individuals).

The vGRFs were measured and recorded through two underbelt force plates (TF-40120-CL and TF-40120-CR, Tec Gihan) at a sampling frequency of 1000 Hz. The vGRF data were filtered using a fourth-order zero-lag low-pass Butterworth filter with cutoff frequency of 20 Hz^31^ and normalized to participant body weight. Stance phase was defined by initial contact and toe-off events as registered using a vGRF threshold of 40 N^32^.

From the instantaneous vGRF data, we extracted.

1. Maximum vGRF during the 0–40% of the stance phase loading period (*F*_z1_)
2. Time between initial foot contact and *F*_z1_ (*t*_z1_) (Fig. 1b).
3. Maximum vGRF in 60–100% of the stance phase unloading period (*F*_z2_)
4. Time between *F*_z2_ and toe-off (*t*_z2_) (Fig. 1b).

Following established protocols^19–22^, the LR was defined as *F*_z1_ divided by *t*_z1_, and the ULR as *F*_z2_ divided by *t*_z2_. Both the LR and ULR were normalized to participant body weight. We assessed the weight transfer strategy by quantifying the following three relationships between the ULR and LR across the different walking speeds in each limb: (1) the relationship between the ULR of the prosthetic limb and LR of the intact limb (ULR_prosthetic__LR_intact_); (2) the relationship between the ULR of the intact limb and the LR of the prosthetic limb (ULR_intact__LR_prosthetic_); (3) the ULR and LR of the control limbs (ULR_control__LR_control_).

Finally, we calculated the double support time defined as the duration from the leading limb’s initial contact event to the trailing limb’s toe–off event. We assessed the double support time for three types of trailing–leading limb orientations (prosthetic–intact, intact–prosthetic, and control–control). The double support time for control participants was assessed as the duration from the leading right limb’s initial contact event to the trailing left limb’s toe–off event.

### Statistical analysis

The Shapiro–Wilk test was used to confirm data normality. As *F*_z1_ and *F*_z2_ were normally distributed, they were tested using a two-way mixed ANOVA (within subject: speeds, between subject: limbs) with Bonferroni test as post-hoc comparison. In contrast, *t*_z1_, LR, *t*_z2_, ULR and double support time were not normally distributed, and thus the Kruskal–Wallis test and Friedman test were used to investigate the main effects of limbs and speeds, respectively. For establishing significance, the Mann–Whitney U test and Wilcoxon signed-rank test were used for post-hoc comparisons considering the limbs and speeds, respectively. Treating the limb data as unpaired in both analyses represents a conservative approach and has been implemented in similar studies^33,34^.

We also performed best-fit linear regression analyses to quantify three relationships of ULR and LR across walking speeds for each participant: (1) ULR_prosthetic__LR_intact_, (2) ULR_intact__LR_prosthetic_, and (3) ULR_control__LR_control_. The third relationship for control participants assessed the correlation between averages of the ULR in the left limb and LR in the right limb. From the three resulting linear regression equations, the slope coefficients, *y*-intercepts, and coefficients of determination (*R*^2^) were calculated. The slope coefficients and y-intercepts reflect the mathematical parameters of the regression lines defining the relationship between LR and ULR. A positive slope coefficient reflects an increasing trend of LR and ULR. When the slope coefficients equal one, the LR and ULR increases with a 1:1 proportion across walking speeds. Moreover, if the slope coefficient is greater than one, the LR increases more than the ULR across speeds. Conversely, if the slope coefficient is less than one, the ULR increases more than the LR. The y-intercept is a measure of bias in the model and basis of predictions for outside the range of observed data. The *R*^2^ values estimate the amount of variability in the relationship accounted for by the regression model, with values approaching one reflecting a better model fit. The slope coefficients, *y*-intercepts, and *R*^2^ compared using the Kruskal–Wallis test and Mann–Whitney U test to investigate the main effects of the relationships and their post-hoc comparisons, respectively. SPSS for Windows Version 26 (IBM, Armonk, NY, USA) was used for all the statistical analyses. The critical α was set at 0.05, with a Bonferroni correction for post-hoc comparisons.

## Results

The mean values of all the limb-related parameters are listed in Table 2. The vGRF from the intact, prosthetic, and control limbs are shown in Fig. 1c–e. In addition, the mean stance time is listed in the Appendix (see Appendix Table S1).

**Table 2:** Mean (standard deviation) values of intact and prosthetic limbs in individuals with UTFA and limbs of control individuals and double support time in each relationship at each experimental walking speed.

		Walking speed							
		2.0 km/h	2.5 km/h	3.0 km/h	3.5 km/h	4.0 km/h	4.5 km/h	5.0 km/h	5.5 km/h
LR (BW/s)	Intact	3.99 (0.90)^$†^	5.01 (1.34)^#$††^	6.14 (1.61)^#$††^	8.09 (1.99)^#$$††^	10.31 (2.34)^#$$††^	12.41 (3.81)^#$$††^	15.41 (4.96)^#$$††^	18.43 (6.06)^#$$††^
	Prosthetic	3.25 (0.61)	3.88 (0.56)^#^	4.66 (0.77)^#^	5.68 (0.73)^#^	7.09 (1.50)^#^	8.70 (2.51)^#^	10.49 (2.80)^#^	12.53 (3.73)^#¶^
	Control	3.18 (0.40)	3.68 (0.46)^#^	4.44 (0.49)^#^	5.26 (0.54)^#^	6.56 (0.80)^#^	7.44 (1.02)^#^	8.37 (1.00)^#^	9.73 (1.35)^#^
Fz_1_ (BW)	Intact	1.03 (0.05)	1.04 (0.05)	1.07 (0.06)^#†^	1.12 (0.08)^##††^	1.21 (0.09)^##$$††^	1.31 (0.11)^##$$††^	1.43 (0.11)^##$$††^	1.55 (0.13)^##$$††^
	Prosthetic	1.05 (0.06)	1.06 (0.04)^¶¶^	1.07 (0.04)^¶^	1.08 (0.05)	1.12 (0.07)^##^	1.18 (0.09)^##^	1.25 (0.10)^##^	1.34 (0.12)^##^
	Control	1.02 (0.02)	1.02 (0.02)	1.02 (0.03)	1.05 (0.04)	1.10 (0.07)^##^	1.14 (0.07)^##^	1.20 (0.08)^##^	1.26 (0.08)^##^
tz_1_ (s)	Intact	0.26 (0.05)^$†^	0.21 (0.05)^#$†^	0.18 (0.04)^#$†^	0.15 (0.03)^#$$††^	0.12 (0.02)^#$$††^	0.11 (0.02)^#††^	0.10 (0.02)^#††^	0.09 (0.02)^#††^
	Prosthetic	0.31 (0.05)	0.26 (0.03)^#^	0.22 (0.03)^#^	0.19 (0.02)^#^	0.15 (0.02)^#^	0.14 (0.02)^#^	0.12 (0.02)^#^	0.11 (0.02)^#¶^
	Control	0.31 (0.04)	0.27 (0.03)^#^	0.22 (0.02)^#^	0.19 (0.02)^#^	0.16 (0.02)^#^	0.15 (0.02)^#^	0.14 (0.01)	0.13 (0.02)^#^
ULR (BW/s)	Intact	2.65 (0.30)	3.26 (0.46)^#^	3.93 (0.74)^#^	5.02 (0.95)^#^	6.03 (1.17)^#^	7.25 (1.21)^#^	8.18 (1.32)^#^	9.18 (1.49)^#^
	Prosthetic	3.57 (0.71)	4.14 (0.77)^#^	4.66 (0.84)^#^	5.25 (0.98)^#^	5.46 (1.02)	5.57 (1.16)	6.03 (1.21)^#^	6.54 (1.37)
	Control	2.92 (0.36)	3.70 (0.46)^#^	4.69 (0.35)^#^	5.38 (0.59)^#^	6.07 (0.94)^#^	6.68 (0.98)^#^	7.54 (1.12)^#^	8.18 (1.06)^#^
Fz_2_ (BW)	Intact	1.00 (0.03)	1.00 (0.03)	1.00 (0.04)	1.01 (0.05)	1.03 (0.07)^#$$^	1.08 (0.09)^##$$^	1.12 (0.12)^##$$^	1.16 (0.14)^##$$^
	Prosthetic	1.00 (0.03)	0.99 (0.03)	0.98 (0.03)^#¶^	0.97 (0.03)^¶¶^	0.96 (0.03)^¶¶^	0.95 (0.03)^¶¶^	0.94 (0.03)^¶¶^	0.93 (0.04)^¶¶^
	Control	0.99 (0.02)	1.00 (0.03)	1.01 (0.04)	1.03 (0.05)^#^	1.04 (0.05)	1.06 (0.06)	1.08 (0.07)^#^	1.10 (0.08)
tz_2_ (s)	Intact	0.36 (0.04)	0.30 (0.04)^#^	0.25 (0.04)^#^	0.20 (0.03)^#^	0.17 (0.02)^#^	0.14 (0.02)^#^	0.13 (0.01)^#^	0.12 (0.01)^#^
	Prosthetic	0.28 (0.05)	0.24 (0.05)^#^	0.21 (0.04)^#^	0.18 (0.04)^#^	0.17 (0.04)	0.17 (0.04)	0.15 (0.03)^#^	0.14 (0.03)^#^
	Control	0.33 (0.04)	0.26 (0.03)^#^	0.21 (0.02)^#^	0.18 (0.02)^#^	0.17 (0.02)^#^	0.15 (0.01)^#^	0.14 (0.01)^#^	0.13 (0.01)^#^
Double support time (s)	Prosthetic–Intact	0.20 (0.04)^$†^	0.17 (0.03)^#$††^	0.15 (0.03)^#$††^	0.13 (0.03)^#$$††^	0.12 (0.02)^#$$††^	0.11 (0.02)^#$$††^	0.10 (0.02)^#$$††^	0.09 (0.02)^#$$††^
	Intact–Prosthetic	0.26 (0.05)	0.20 (0.03)^#^	0.17 (0.02)^#^	0.15 (0.02)^#^	0.13 (0.02)^#^	0.12 (0.02)^#^	0.10 (0.02)^#^	0.09 (0.01)^#¶¶^
	Control–Control	0.24 (0.03)	0.20 (0.02)^#^	0.17 (0.02)^#^	0.15 (0.02)^#^	0.13 (0.02)^#^	0.12 (0.02)^#^	0.11 (0.01)^#^	0.10 (0.01)^#^

^#,##^indicate significant differences between the current and previous speeds at *P* < 0.05 and *P* < 0.01, respectively.

^$,$$^indicate significant differences between the values in the intact and prosthetic limbs or the prosthetic–intact and intact–prosthetic relationships at *P* < 0.05 and *P* < 0.01, respectively.

^†,††^indicate significant differences between the values in the intact and control limbs or the prosthetic–intact and control–control relationships at *P* < 0.05 and *P* < 0.01, respectively.

^¶^,^¶¶^indicate significant differences between the values in the prosthetic and control limbs or the intact–prosthetic and control–control relationships at *P* < 0.05 and *P* < 0.01, respectively. BW, body weight.

### Loading rate and the first peak of vertical ground reaction force

Significant main effects of limbs and speeds were observed on the LR (limb: *P* < 0.001, speed: *P* < 0.001). The intact limb LR was significantly greater than for the prosthetic and control limbs for all walking speeds (*P* ≤ 0.048). Moreover, the intact LR was on average 39% and 56% greater than the prosthetic and control limbs, respectively, across walking speeds. The prosthetic limb LR was significantly greater than for control limbs only at 5.5 km/h (*P* = 0.048) and was on average 12% greater than the control limbs across walking speeds. For all limbs, the LR significantly increased with increasing walking speed (*P* ≤ 0.028). The LR in the intact, prosthetic and control limbs increased from 2.0 to 5.5 km/h by 362%, 285% and 206%, respectively. There were also significant main effects of limbs and speeds, as well as a significant interaction effect, on *F*_z1_ (limb: *P* < 0.001, speed: *P* < 0.001, interaction: *P* < 0.001). The *F*_z1_ magnitude for the intact limb was significantly greater than that in the prosthetic limb for 4.0–5.5 km/h (*P* ≤ 0.009) and control limbs for 3.0–5.5 km/h (*P* ≤ 0.022). The *F*_z1_ in the intact limb was on average 6% and 10% greater than that in the prosthetic and control limbs, respectively, across walking speeds. Furthermore, *F*_z1_ in the prosthetic limb was significantly greater than that in the control limb at 2.5 and 3.0 km/h (*P* ≤ 0.029) and was on average 4% greater than the control limbs across walking speeds. *F*_z1_ in the intact limb significantly increased at 3.0 km/h and higher speeds (*P* ≤ 0.027), whereas the significant increase for the prosthetic limb and control limbs occurred from 4.0 km/h (*P* ≤ 0.008). *F*_z1_ in the intact, prosthetic and control limbs increased between 2.0 to 5.5 km/h by 51%, 28% and 23%, respectively.

### Unloading rate and the second peak of vertical ground reaction force

No significant main effect of limbs was observed in the ULR, but a significant main effect of speed was revealed in the ULR (*P* < 0.001). The ULR in the intact and control limbs increased with increasing walking speed (*P* ≤ 0.034) and the ULR in the prosthetic limb increased for 2.0–3.5 km/h and 4.5–5.0 km/h (*P* ≤ 0.043). The ULR in the intact, prosthetic and control limbs increased between 2.0 to 5.5 km/h by 247%, 83% and 180%, respectively. We also observed significant main effects of limbs and speed, as well as a significant interaction effect, on *F*_z2_ (limb: *P* < 0.001, speed: *P* < 0.001, interaction: *P* < 0.001). The *F*_z2_ magnitude in the intact limb was significantly greater than that in the prosthetic limb for 4.0–5.5 km/h (*P* ≤ 0.005). While the *F*_z2_ in the intact limb was on average 9% greater than that in the prosthetic limb across walking speeds, the difference between intact and prosthetic limbs became greater at faster walking speeds. Furthermore, *F*_z2_ in the control limb was significantly greater than that in the prosthetic limb for 3.0–5.5 km/h (*P* ≤ 0.048). The *F*_z2_ in the control limb was on average 8% greater than that in the prosthetic limb across walking speeds and the difference between prosthetic and control limbs also became greater at faster walking speeds. However, no significant difference was observed between the intact limb and control limbs at any speed. Although *F*_z2_ increased in the intact and control limbs with increasing walking speed, its value in the prosthetic limb remained mostly constant or even decreased with increasing walking speed.

### The relationships of unloading rate and loading rate

**Figure 2:**
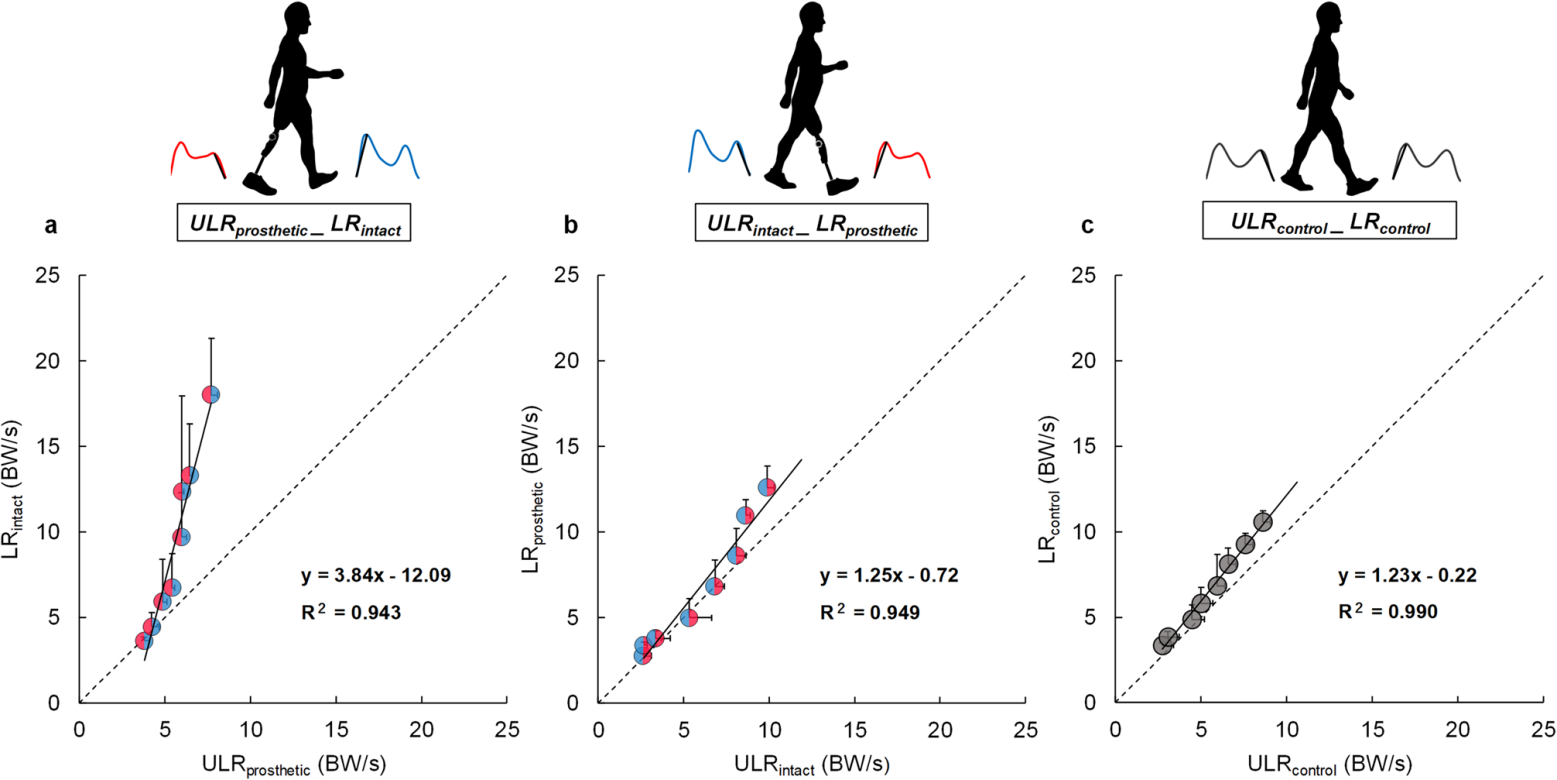
Relationships between ULR and LR: (**a**) ULR_prosthetic__LR_intact_, (**b**) ULR_intact__LR_prosthetic_, and (**c**) ULR_control__LR_control_ from representative participants. Each circle represents one of eight different walking speeds, respectively. Dotted lines indicate the identity line, where the ULR and LR have the same slope. Figure was created in Microsoft Power Point for Office 365 MSO (16.0.12527.21378).

**Figure 3:**
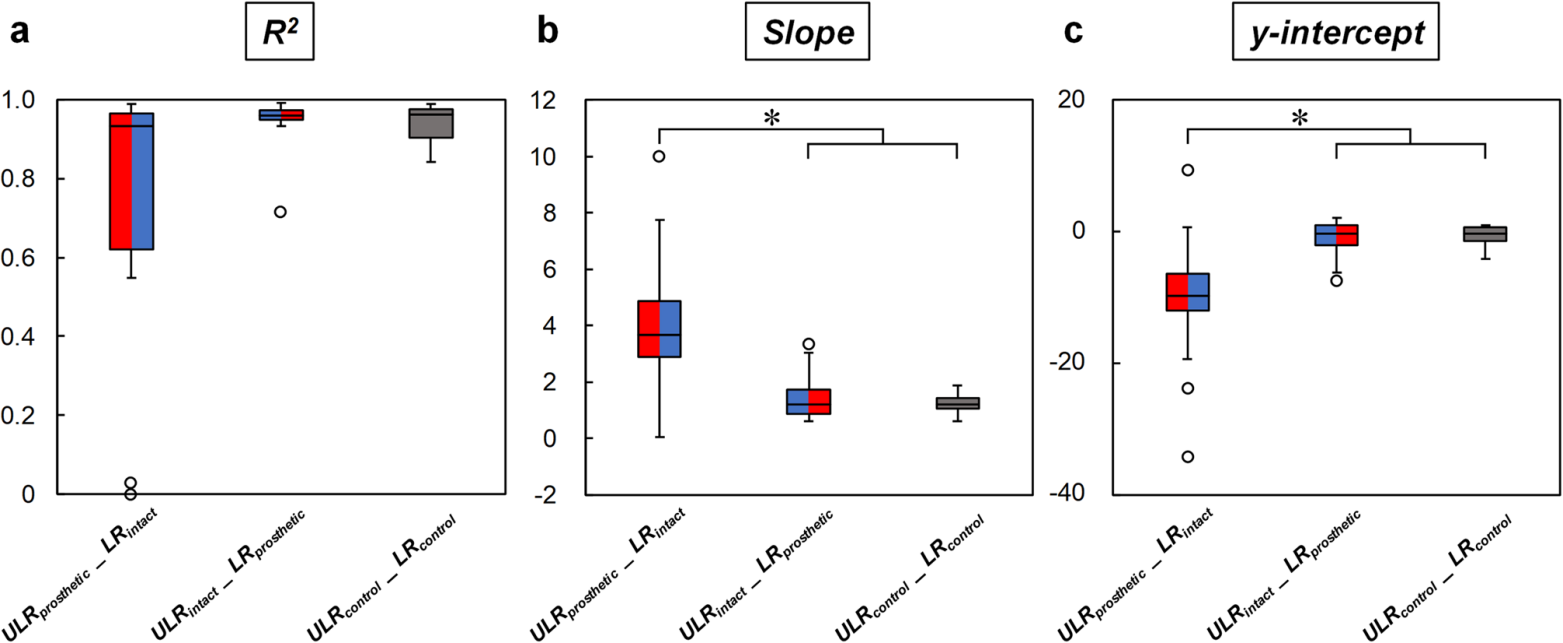
Comparison of (**a**) *R*^2^, (**b**) slope, and (**c**) *y*-intercept of relationships ULR_prosthetic__LR_intact_ (red and blue) ULR_intact__LR_prosthetic_ (blue and red) and ULR_control__LR_control_ (gray). The box plots indicate the median values, interquartile range, and outliers. The open circles indicate outliers in each relationship. The asterisks (*) indicate significant differences (*P* < 0.05) between the corresponding relationships.

Representative examples of the relationships between the ULR and LR obtained from best-fit linear regressions for one participant are shown in Fig. 2a–c. The *R*^2^, slope coefficients and *y*-intercepts for all estimated relationships are shown in Fig. 3a–c. We found that there was no significant main effect on *R*^2^ across relationships among ULR_prosthetic__LR_intact_, ULR_intact__LR_prosthetic_, and control limb’s ULR_LR (Fig. 3a). Thus, the linear best fit captured equal variance for each relationship, and was equally valid. A significant main effect of the relationship type was observed for the mean slope coefficient (*P* < 0.001), with the coefficient of ULR_prosthetic__LR_intact_ being significantly greater than for the ULR_intact__LR_prosthetic_ and ULR_control__LR_control_ (Fig. 3b; *P* < 0.001). However, no significant difference was observed in the mean slope coefficient between ULR_intact__LR_prosthetic_ and ULR_control__LR_control_. We also found a significant main effect of the relationship type on the *y*-intercept (Fig. 3c; *P* < 0.001). The absolute mean *y*-intercept for ULR_prosthetic__LR_intact_ was significantly greater than for ULR_intact__LR_prosthetic_ and ULR_control__LR_control_ (*P* ≤ 0.001). No significant difference was observed in the mean *y*-intercept between ULR_intact__LR_prosthetic_ and ULR_control__LR_control_.

### Double support time

Significant main effects of limb (i.e., trailing-leading orientation) and speed were observed for double support time (limb: *P* < 0.001, speed: *P* < 0.001). The double support time for the prosthetic–intact orientation was significantly shorter than for the intact–prosthetic orientation and control limbs for all walking speeds (*P* ≤ 0.048). The double support time in the intact–prosthetic orientation was significantly shorter than for the control limbs only at 5.5 km/h (*P* = 0.048). However, the double support time in the intact–prosthetic and control limbs were equivalent across all speeds. For all limbs, the double support time significantly decreased with increasing walking speed (*P* ≤ 0.028).

## Discussion

The aim of this study was to investigate the weight transfer strategy between intact and prosthetic limbs at different walking speeds in individuals with UTFA, and compare this behavior to healthy controls. Specifically, ULR_prosthetic__LR_intact_ noticeably deviated from the identity line (i.e., equal rates proportional with speed) and the other two relationships (Fig. 2a), whereas the ULR_intact__LR_prosthetic_ profile resembled that of ULR_control__LR_control_ (Fig. 2b,c). These results support our initial hypothesis that the ULR_LR relationships would depend on which limb of UTFAs are in the trailing and leading positions.

The mean slopes and absolute *y*-intercept of the best-fit linear regression for the ULR_prosthetic__LR_intact_ relationship was significantly greater than those for ULR_intact__LR_prosthetic_ and ULR_control__LR_control_, respectively (Fig. 3b,c). Hence, although the LR in the intact limb increased substantially, the ULR in the prosthetic limb increased slightly with increasing walking speed. These results suggest that the intact limb is exposed to a higher LR when the trailing limb is the prosthetic limb. These results partially support our hypothesis that the ULR_LR relationships in individuals with UTFA would deviate with increasing walking speeds from that in controls. The results may be partially explained by limited restorative forces produced by the trailing prosthetic limb due to the capabilities of the prosthetic knee and foot. While there was no significant difference between ULR in the three types of limbs, the increasing trends of ULR with increasing walking speed were different. The ULR in the intact and control limbs increased with increasing walking speed by 247% and 180% at 2.0 to 5.5 km/h, respectively, but that in the prosthetic limb increased by only 83% (Table 2). However, *F*_z2_ in the prosthetic limb was significantly less than that in the intact and control limbs over a wide range of walking speeds (Table 2). Furthermore, unlike the intact limb and control limb, *F*_z2_ in the prosthetic limb remained nearly unchanged and even decreased with increasing walking speed (Table 2). This trend is consistent with a previous paper that observed mechanical work in prosthetic limb during push-off to be less than that in intact and control limbs^27^. The relatively low and walking speed-invariant *F*_z2_ is often observed in pathological gait^35^. A lower *F*_z2_ in the prosthetic limb has been related to insufficient ankle push-off in the late stance phase, because *F*_z2_ is predominantly produced through ankle plantarflexion^36^. In addition, a lower *F*_z2_ in the prosthetic limb may be associated with the shorter double support time when the prosthetic limb is in the trailing position (Table 2). This shorter double support time may indicate that weight transfer from the prosthetic to intact limb occurs more rapidly than for the opposite orientation. Consequently, insufficient ankle plantarflexion during push-off of the trailing prosthetic limb that is not restored through hip power may be responsible for the increased LR of the leading intact limb.

The LR of the leading limb may be partially affected by the ULR of the trailing limb. In this study, we found no significant differences in the mean slopes and *y*-intercept between the ULR_intact__LR_prosthetic_ and ULR_control__LR_control_ relationships (Fig. 3b,c). These results may be due to the trailing function of the intact limb, which retains the same physiological mechanisms as the control limb to generate push-off power and regulate change in body center of mass velocity^37^. To this effect, we found no significant difference in *F*_z2_ between the intact and control limbs regardless of the speed (Table 2), and this magnitude increased with increasing walking speed as expected (Table 2). This trend is consistent with the previous study that mechanical work in the intact and control limbs increased with increasing walking speed^27^. Furthermore, while the double support time when the prosthetic limb is in the trailing position was shorter at all speeds, there was also no difference between the double support time for the opposite limb orientation and control limbs at almost all speeds (Table 2). These results indicate that weight transfer from the intact to prosthetic limb or between control limbs takes more time than that from prosthetic to intact limbs. Consequently, the ULR is similar between the intact and control limbs at different walking speeds. In addition, we found no significant difference in *R*^2^ between the three ULR_LR relationships (Fig. 3a). This result indicates the linear correlation between the ULR and LR regardless of each relationship, even if the ULR_prosthetic__LR_intact_ deviated from other two relationships. Thus, the trailing limb unloading during push-off may be related to the leading limb loading following initial contact. The comparisons between the different relationships indicate the importance of jointly evaluating the trailing limb unloading during push-off and the leading limb loading following initial contact in the analysis of weight transfer strategies.

Evidence suggests that high peak loads during the load acceptance phase of stance in the intact limb, as observed in this study relative to the prosthetic and control limb, may result in secondary musculoskeletal injury in individuals with lower limb amputation^11,38–40^. Previous studies also suggest increased LR may lead to secondary musculoskeletal injury^24,25^. In this study, we found that the mean slopes of relationship ULR_prosthetic__LR_intact_ were significantly greater than those of relationships ULR_intact__LR_prosthetic_ and ULR_control__LR_control_ (Fig. 3b), suggesting that the leading intact limb is exposed to a higher risk of secondary musculoskeletal injury during trailing with the prosthetic limb. On the other hand, active plantarflexion produced by a powered ankle–foot prosthesis in individuals with transtibial amputation during late stance decreases *F*_z1_ and the subsequent LR in the leading intact limb^41,42^. Additionally, prosthetic knee joint mechanical function^43,44^, residual hip physiological function^27^ or any combination of these variables may also affect the GRF during walking. Therefore, improving the prostheses functions or movement in the trailing prosthetic limb may reduce the risk of secondary musculoskeletal injury in the intact limb.

There are several limitations that should be considered when interpreting the results of this study. First, we did not control the types of prosthetic components among the participants with UTFA. As shown in previous studies, the types of prosthetic knees^43^, prosthetic alignment^45^ and suspension system^46^ could influence the vGRF during walking. Thus, caution needs to be taken regarding the interpretation and generalization of these findings. Second, participants with UTFA were relatively young (30 ± 9 years), none were of vascular etiology, and there was a broad range of time since amputation (1–32 years). According to previous studies, vGRF features may be influenced by the amputation etiology^47^, adaptation to prosthesis use^43^ and time since discharge from acute rehabilitation^48^. Hence, investigating the ULR_LR relationships in individuals of an improved representative sample may provide additional insights into weight transfer strategies in this cohort. Third, the relationship between ULR and LR may be affected by spatiotemporal constraints during walking. In fact, a previous study reported that the LR was correlated with cadence and stride length in healthy participants^23^, suggesting that cadence and step length could be potential confounders for LR. Therefore, other potential moderating variables affecting LR should be examined in future studies. Fourth, we did not address the timing of the ULR and LR beyond reporting the time between the peaks and the gait events. An examination of the temporal sequencing between events in future work might be helpful for creating a more comprehensive understanding of inter-limb coordination. Finally, as this study was focused on level walking, the ULR_LR relationship in individuals with UTFA may be dependent on locomotor scenario, including slope walking^22^, stair walking^21^, and obstacle crossing^48^. Especially, the negative y-intercept suggest that our model may not cover the small ULR situation such as very slow walking^49^. Future studies should investigate weight transfer between limbs in individuals with UTFA while performing different activities of daily living.

## Conclusion

We investigated the weight transfer strategy between intact and prosthetic limbs at different walking speeds in individuals with UTFA. When the trailing and leading limbs were the prosthetic and intact limbs, respectively, the weight transfer strategy of individuals with UTFA was different than individuals without amputation. Conversely, the weight transfer strategy of individuals with UTFA was similar to that of individuals without amputation across walking speeds when the trailing and leading limbs were the intact and prosthetic limbs, respectively. Results suggest that the weight transfer strategy is varied when leading or trailing limbs are the prosthetic or intact limb, and that the LR of the leading limb may be partially affected by the ULR of the contralateral trailing limb. As an adequate weight transfer strategy is required for successful gait rehabilitation in individuals with UTFA, the simultaneous evaluation of both the trailing limb unloading and leading limb loading are important for fully characterizing motor strategies for forward ambulation in prosthesis users and designing targeted rehabilitation interventions.

## Supplementary Information

Supplementary Information 1.

Supplementary Information 2.
