# Supplementary material for: Inter-limb weight transfer strategy during walking after unilateral transfemoral amputation
Source: Sci Rep. 2021 Feb 26;11:4793. doi: 10.1038/s41598-021-84357-9 (PMC7910552; doi:10.1038/s41598-021-84357-9)
Supplement: Supplementary file 1 — Supplementary Information 1. [file 41598_2021_84357_MOESM1_ESM.docx]

**Supplementary material**

**Table S1.** Mean (standard deviation) values of stance time in individuals with UTFA and limbs of control individuals at each experimental walking speed. The stance time was defined as the duration from initial contact to toe–off. The Kruskal–Wallis test and Friedman test were used to investigate the main effects of limbs and speeds, respectively. The Mann–Whitney U test and Wilcoxon signed-rank test were used for post-hoc comparisons considering the limbs and speeds, respectively. # indicates significant differences between the current and previous speeds at *P* < 0.05. $ and $$ indicate significant differences between the values in the intact and prosthetic limbs at *P* < 0.05 and *P* < 0.01, respectively. ¶ and ¶¶ indicate significant differences between the values in the prosthetic and control limbs at *P* < 0.05 and *P* < 0.01, respectively.
